# Effects of Mindfulness-Based Interventions on Salivary Cortisol in Healthy Adults: A Meta-Analytical Review

**DOI:** 10.3389/fphys.2016.00471

**Published:** 2016-10-19

**Authors:** Kenji Sanada, Jesus Montero-Marin, Marta Alda Díez, Montserrat Salas-Valero, María C. Pérez-Yus, Héctor Morillo, Marcelo M. P. Demarzo, Mauro García-Toro, Javier García-Campayo

**Affiliations:** ^1^Primary Care, Aragon Health Sciences InstituteZaragoza, Spain; ^2^Department of Psychiatry, Showa University School of MedicineTokyo, Japan; ^3^Faculty of Health Sciences and Sports, University of ZaragozaZaragoza, Spain; ^4^The Primary Care Prevention and Health Promotion Research NetworkBarcelona, Spain; ^5^Department of Psychiatry, Miguel Servet University Hospital, University of ZaragozaZaragoza, Spain; ^6^Department of Preventive Medicine, “Mente Aberta” Brazilian Centre for Mindfulness and Health Promotion, Federal University of São PauloSão Paulo, Brazil; ^7^Hospital Israelita Albert EinsteinSão Paulo, Brazil; ^8^Research Institute of Health Sciences, University of the Balearic IslandsPalma, Spain

**Keywords:** MBI, salivary cortisol, healthy adult subjects, RCT, meta-analysis

## Abstract

**Objective:** The aim of the present study was to elucidate the effects of Mindfulness-based interventions (MBIs) on salivary cortisol levels in healthy adult populations.

**Method:** We conducted a systematic review and meta-analysis of randomized controlled trials (RCTs), published between January 1980 and June 2015 in PubMed, EMBASE, PsycINFO and the Cochrane library. The PRISMA and Cochrane guidelines were followed. The pooled effect sizes were calculated with the random-effects model, using Hedges' *g*-values, and heterogeneity was measured using the *I*^2^ statistic. The contribution of different characteristics of participants and programmes were assessed by meta-regression models, using beta coefficients.

**Results:** Five RCTs with 190 participants in total were included in this systematic review. The overall effect size (ES) for improving the state of health related to cortisol levels was moderately low (*g* = 0.41; *p* = 0.025), although moderate heterogeneity was found (*I*^2^ = 55; *p* = 0.063). There were no significant differences between active (*g* = 0.33; *p* = 0.202) and passive (*g* = 0.48; *p* = 0.279) controls, but significant differences were found when comparing standard (*g* = 0.81; *p* = 0.002) and raw (*g* = 0.03; *p* = 0.896) measures. The percentage of women in each study was not related to ES. Nevertheless, age (beta = −0.03; *p* = 0.039), the number of sessions (beta = 0.33; *p* = 0.007) and the total hours of the MBI (beta = 0.06; *p* = 0.005) were significantly related to ES, explaining heterogeneity (*R*^2^ = 1.00).

**Conclusions:** Despite the scarce number of studies, our results suggest that MBIs might have some beneficial effect on cortisol secretion in healthy adult subjects. However, there is a need for further RCTs implemented in accordance with standard programmes and measurements of salivary cortisol under rigorous strategies in healthy adult populations.

## Background

In recent years, many articles on the subject of meditation, and more specifically mindfulness-based interventions (MBIs), have been published in rapid succession. Although there are presently different types of MBIs with specific psycho-educational components adapted to the target populations, their roots can be traced back to the late 1970's. A mindfulness-based stress reduction (MBSR) programme was begun in 1979 in the basement of the University of Massachusetts Medical Center (Cullen, 2011). That was where Kabat-Zinn (1982) initially reported that mindfulness meditation showed significant pain reduction in chronic pain patients. Since then, the numerous treatment protocols based on MBSR, such as mindfulness-based cognitive therapy (MBCT), have been developed. In particular, MBSR and MBCT are two of the most widely used MBIs (O'Leary et al., 2016).

In general, MBIs combine meditation practices with stress reduction programmes and contemporary cognitive-behavioral approaches (Cullen, 2011). Their positive effects on mental health and quality of life have been reported in diverse clinical and non-clinical populations (Khoury et al., 2013; Goyal et al., 2014; Demarzo et al., 2015). However, not many articles have examined the relationship between MBIs and biomarkers. The most frequently studied biomarker featured in these studies is cortisol. Cortisol is a steroid hormone released by the adrenal cortex in response to stress levels. It is accepted as an objective biological marker of stress, and is relatively accessible to clinical researchers (Matousek et al., 2010). Cortisol measurement can be performed on plasma/serum, urine, saliva, and hair. Among these, the analysis of salivary cortisol has several advantages over that of blood cortisol (e.g., stress-free sampling, laboratory independence, low costs; Kirschbaum and Hellhammer, 1994). In general, salivary measures of cortisol are considered a valid and reliable alternative to measuring free cortisol in serum (Matousek et al., 2010).

There was only one previous systematic review conducted with a focus on addressing mindfulness intervention effects on salivary cortisol, whereas findings were inconsistent across included studies (O'Leary et al., 2016). That review included not only healthy subjects, but also participants with substance abuse, breast cancer, depression and overweight/obesity, and as a consequence, it seemed to be limited to interpreting the effects on cortisol levels owing to the high heterogeneity of the included samples. It is true that mindfulness interventions have been used both to treat medical and psychiatric disorders (i.e., to decrease negative affect), and to improve psychological well-being in healthy people (i.e., to increase positive affect). However, it is not clear that the effect of this kind of therapy is exactly the same in both populations, and therefore it seemed recommendable to be stringent with one study target (Demarzo et al., 2015). For this reason, we focused only on healthy populations. Thus, the aim of the present systematic review and meta-analysis was to explore the efficacy of MBIs on salivary cortisol in healthy adults.

## Methods

The PRISMA guidelines for systematic reviews and meta-analyses (Shamseer et al., 2015) and the recommendations of the Cochrane Collaboration were followed (Higgins et al., 2011). The protocol was registered with the International Prospective Register of Systematic Reviews (PROSPERO), under registration number CRD42016035297.

### Eligibility criteria

The study eligibility criteria are shown in Table 1. No restrictions were applied regarding comparator characteristics (waiting list, or any active control group, including other types of psychosocial interventions), follow-up or type of data analysis.

**Table 1:** **Study eligibility criteria**.

	**Inclusion criteria**	**Exclusion criteria**
Participants	Healthy adult subjects (aged ≥18 years).	Patients with some kind of diseases, pregnancy, and obesity.
	No restrictions regarding the number of participants.	
Interventions	Mindfulness-based interventions (MBIs).	Other non-pharmacological interventions.
	With a minimum duration of 6 weeks.	
Outcome	At least salivary cortisol outcomes in normal conditions (without a stress test).	Only other biomarkers, or only stress test assessments.
Study design	RCTs.	Non-RCTs, open trials with a pre-post analysis.
Publications	Published in English, French, or Spanish and as full-text articles in peer-reviewed scientific journals from January 1980 to May 2015.	Published in other languages and as reviews, case reports or letters.

Non-RCTs, non-randomized controlled trials; RCTs, randomized controlled trials.

### Search strategy

An exhaustive systematic literature search, using PubMed, EMBASE, PsycINFO, and the Cochrane library, was conducted by an expert in this field (MSV), on studies published between January 1980 and June 2015. The starting date was set because the first paper on MBSR was published in 1982 (Kabat-Zinn, 1982). The search terms for the PubMed database can be seen in Table 2. Search results were imported into an electronic bibliography after the removal of duplicated citations. The reference lists of the identified original articles and reviews were also screened manually, and other experts in the field were also contacted for identification of additional studies. The last search was conducted on 14 July 2015.

**Table 2:** **Search terms for the PubMed database**.

**((“Mindfulness”[Mesh] OR mindfulness OR “mindfulness meditation” OR “meditation” OR “mindfulness based cognitive therapy” OR MBCT OR “mindfulness based stress reduction” OR MBSR)) AND (cortisol)**.

### Data extraction and coding procedure

Two authors (KS and MCPY) independently screened the titles and abstracts retrieved from the electronic databases and independently assessed the full texts of each study. Any disagreements were resolved by discussion and consensus, and where doubts remained, the final decision was made in consultation with other authors (MMPD and JGC). The identified literature was coded and the data extracted, using a predefined data extraction sheet, for the following items: year of publication, number of participants, sample origin, mean age, percentage of women in the sample, MBI type, and characteristics (number of sessions, total weeks, hours of intervention, contents of homework), type of control group [active control (AC) or passive control (PC)], study duration, intention-to-treat analysis, salivary cortisol assessments (total times and periods of measures during study and in a day of measures), and other outcomes.

### Assessment of study quality

Risk of bias was assessed with four criteria from the Cochrane Collaboration's tool (Higgins et al., 2011): adequate generation of allocation sequence, concealment of allocation to conditions, prevention of knowledge of the allocated intervention, and dealing with incomplete outcome data. We considered those studies that met three or more criteria as high quality, and those that met fewer criteria as low quality (Cuijpers et al., 2014). Assessment of quality was independently performed by two reviewers (KS and HM), and any divergences were resolved through discussion or consultation with other reviewers (MCPY and JGC). The quality of the interventions was evaluated according to three criteria from an authoritative review of empirically supported psychotherapies (Chambless and Hollon, 1998): (1) the study referred to the use of a treatment manual; (2) the therapy was provided by specifically trained therapists; and (3) treatment integrity was verified during the study. Two reviewers (KS and HM) independently assessed these criteria, and any discrepancies were discussed with a third reviewer (MCPY) for consensus.

### Data synthesis

Measurements were mainly collected from the outcomes of standardized salivary cortisol indices, such as: cortisol awakening response (CAR), daily output and diurnal slope. CAR is defined as the change in cortisol concentration that occurs during the first hour after waking from sleep (Clow et al., 2004), and it was calculated for morning samples using the area under the curve with respect to ground (AUC_G_), or with respect to increase from awakening (AUC_I_). AUC is one of methods for analysing the overall secretion over a specific time-period in endocrinologic studies, and there are two formulas for calculating it, referred to as AUC_G_ and AUC_I_ (Pruessner et al., 2003). Daily output was calculated using the area under the curve with respect to ground (AUC_G_) during a whole day. Diurnal slope is also one of the methods for analysing cortisol concentrations focused on the diurnal cycle, where the levels of cortisol are high in the morning and low at night. On the other hand, we also included morning levels and average daily values as raw data outcomes. The morning cortisol level was assessed using the peak level at 30 min after awakening as CAR without correction for baseline differences, and average daily values were calculated as the mean levels of each measured value in a day. Generally, daily output and diurnal slope are considered indicators that reflect basal secretion, while CAR is an index that reflects reactivity in response to stimulation (Izawa et al., 2010). CAR has been said to differ from total daily cortisol exposure (Golden et al., 2013).

Although it is not free from controversy, in general, higher morning levels and diurnal slope values are considered to indicate a better health status (Sephton et al., 2000; Adam and Gunnar, 2001; Keller et al., 2006; Adam and Kumari, 2009; Hsiao et al., 2010, 2012; Stawski et al., 2011; Carlson et al., 2013), while higher daily output (AUC during the whole day) and average daily values are considered indicators of worse health status (Brown et al., 2004; Steptoe et al., 2005; Chan et al., 2006; Lovell et al., 2011). It has been said that job stress and general life stress are associated with an increased CAR, and therefore the higher the CAR, the worse the states of health (Chida and Steptoe, 2009). However, this same work (Chida and Steptoe, 2009), also demonstrated associations of low CAR with fatigue and burnout. We must stress that we selected studies of healthy subjects for this analysis, and because of this, only the associations between cortisol and health in healthy participants are relevant, which somewhat reduces possible variation and inconsistency.

We took into account the post-test measurements which were collected immediately after the intervention time, as well as all the follows-up used (we found 4 months maximum). The effect size (ES), indicating the differences between the two groups, and 95% confidence intervals (CIs) were calculated (Hedges and Olkin, 1985; Cooper and Hedges, 1994). Whenever necessary, combined outcomes were estimated using a pooled mean ES provided by the Comprehensive Meta-Analysis-3.0 computer program.

Hedges' g was chosen as the ES measure, since the present meta-analysis includes studies with a small sample size, and this measure adjusts accordingly (Hedges, 1981). It has been suggested that 0–0.40 can be considered small; 0.4–0.7, moderate; and 0.7 and above, large (Higgins and Green, 2008). The pooled ESs were calculated with the random effects model. Because of the difficulty in interpreting Hedges' g from a clinical point of view, we also converted these values into the number needed to treat (NNT), according to Furukawa's formula (Furukawa and Leucht, 2011). NNT indicates the number of participants who need to be treated in order to generate one additional, clinically significant, favorable change (Laupacis et al., 1988), and points out effective treatments usually in the range of 2–4. We tested heterogeneity using the *I*^2^ statistic and 95% CI when possible, assuming a value of around 25% to indicate low heterogeneity; 50%, moderate; and 75%, high (Hedges and Vevea, 1998; Higgins et al., 2003; Borenstein et al., 2009; Wersebe et al., 2013). We also calculated the Q statistic and the associated *p*-value. A significant *p*-value (< 0.05) indicates the presence of heterogeneity.

Subgroup analyses were conducted with the mixed effects model to evaluate possible differences according to the comparison group, active control (AC) vs. passive control (PC), and outcome used, standard indices vs. raw data. This analytical model pools studies within the subgroups of the random effects model and tests for significant differences between subgroups with the fixed effects model (Cuijpers et al., 2011). A meta-regression analysis was also developed, taking separately the percentage of female participants, age, number of MBI sessions and hours of programme as independent variables, through the use of beta coefficients (and CIs) in mixed-effects models. *R*^2^ was calculated to assess the proportion of total between-study variance explained by the meta-regression models, and their goodness of fit was estimated assuming that the unexplained variance was null.

Publication bias was assessed initially through the construction of a funnel plot analysis (Duval and Tweedie, 2000; Vevea and Woods, 2005; Cuijpers et al., 2008). Egger's test was used to contrast the null hypothesis with biased absences (Egger et al., 1997), and Duval and Tweedie's trim and fill procedure (Duval and Tweedie, 2000) provided the number of studies that were probably absent, allowing an estimate of the ES taking publication bias into account. The Begg and Mazumdar rank correlation test was also applied to test whether the adjusted and observed ESs differed significantly from each other (Begg and Mazumbar, 1994).

All of the tests were bilateral and were performed with a significance level of *p* < 0.05, except for the bias-related tests, which were unilateral.

## Results

**Figure 1:**
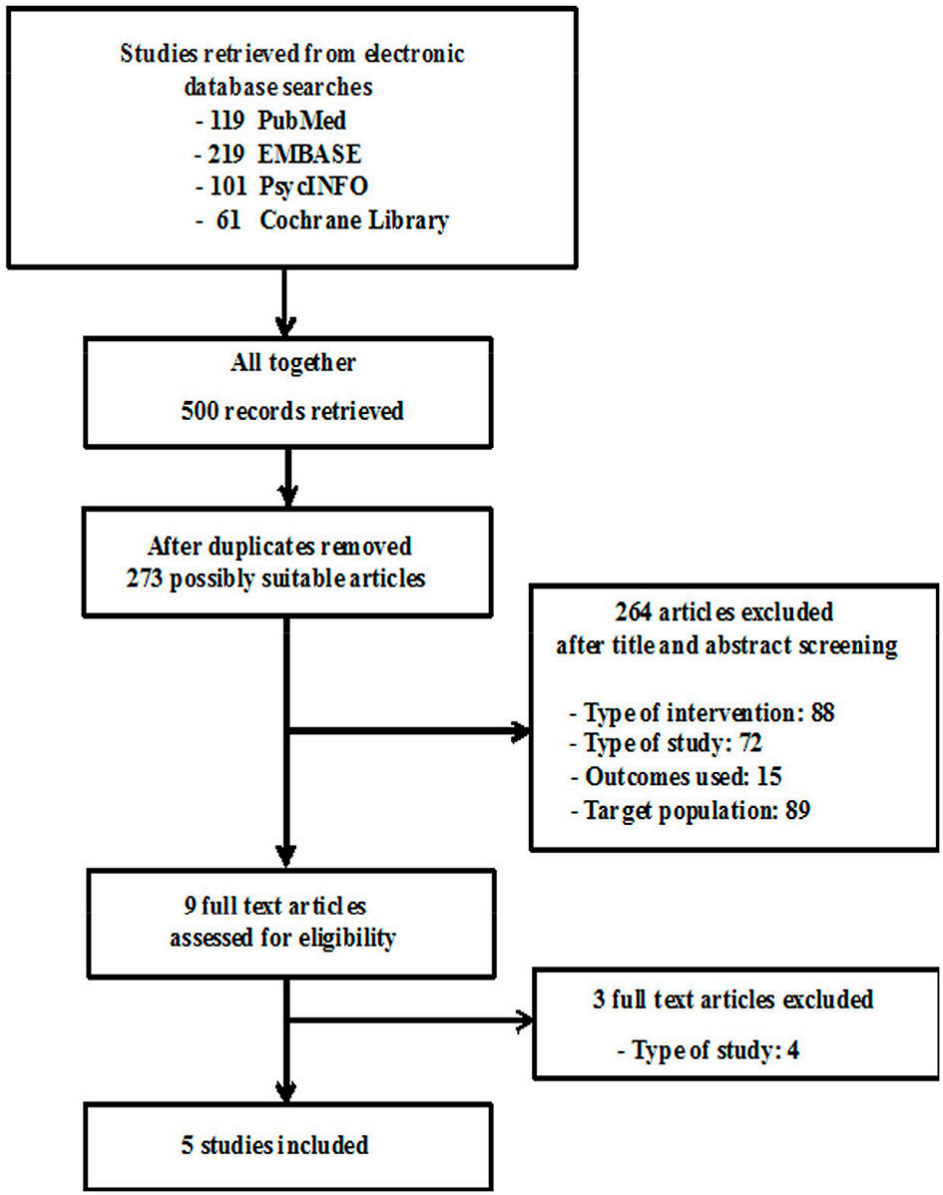
**Algorithm of study selection**.

Of the initial search of 500 records, including 227 duplicates, 264 were excluded after title and abstract screening, and 9 articles were assessed as full text (Figure 1). There were four main reasons for excluding articles: (1) the study was not related to the targeted intervention; (2) the study was not performed with RCTs; (3) the study did not examine the levels of salivary cortisol in normal conditions, in other words, it only assessed cortisol under a stress test condition; (4) the target population did not consist of healthy subjects. After a full reading of the texts, we finally included five articles with a total of 190 participants (86 of whom were treated with some kind of MBI).

Given the effects of some confounding factors on the levels of salivary cortisol (e.g., pregnancy, obesity), we excluded six trials after examining the possible effects of participants' characteristics (Beddoe et al., 2009; Daubenmier et al., 2011, 2012; Chan, 2014; Mason et al., 2015; Zhang and Emory, 2015). Two trials were also excluded because they were conducted on school children (Sibinga et al., 2013; Schonert-Reichl et al., 2015); along with a further two systematic reviews taking into account MBIs with adolescents (Zoogman et al., 2015; Felver et al., 2016), and another two trials which were performed with substance abusers (Marcus et al., 2003) and with a C-reactive protein (CRP) level >3 mg/ml (Malarkey et al., 2013). In relation to interventions and outcomes, one trial carried out with a brief 3-day MBI was dismissed (Creswell et al., 2014). Three trials investigated other materials for cortisol, such us serum (Daubenmier et al., 2012; Kim et al., 2013) and hair (Goldberg et al., 2014), and one study only used a stress test procedure to measure cortisol, with no comparable results (Nyklíček et al., 2013). Regarding study designs, we excluded non-randomized controlled trials (non-RCTs; i.e., Lynch et al., 2011; Ramler et al., 2015) and open trials with a pre-post analysis (Galantino et al., 2005; Brand et al., 2012; Ruiz-Robledillo et al., 2015; Christopher et al., 2016).

### Characteristics of included studies

The characteristics of all five included RCTs are shown in Table 3. Of the included RCTs, two studies (Oken et al., 2010; Jensen et al., 2012) were conducted with a two-arm control design (ACs and PCs); another two (Klatt et al., 2009; Flook et al., 2013) were conducted with PCs; and the remaining one (Rosenkranz et al., 2013) was conducted with an AC. There was only one study (Rosenkranz et al., 2013), that included follow-up after intervention (4 months). With regard to participants, the mean age ranged from 38.50 to 67.09 (mean: 44.50; *SD*: 14.36). The sample size ranged from 18 to 49 (average: 38; *SD*: 13.23). The mean proportion of women in the samples was 78.4% (*SD*: 8.38), and ranged from 66 to 89. By paying attention to each included study from the perspective of stress, it was found that the participants of one trial (Oken et al., 2010) were under possible high-stress baseline conditions, as they were caregivers who spent at least 12 h per week providing assistance for close relatives with dementia.

**Table 3:** **Characteristics of studies**.

**References**	**Population**	**MBI program**	**Controls**	**Study duration**	**Intention-to-treat**	**Cortisol assessments**	**Cortisol indicators**	**Other biomarkers**	**Results**	**St Q**	**Tx Q**
Klatt et al., 2009	45 university employees Mean age: 43.41 ± 2.17 years (MBSR), 46.50 ± 1.89 years (Control) Gender: male 24%, female 76% (total).	MBSR-id (low-dose), (*N* = 22), 6 weeks programme training of 6 h. Homework: individual sessions for 20′ during the remaining work days. Listen to the daily meditations on the CD four times per week. Adherence workbook and two CDs.	Wait-list control (PC) (*N* = 23).	N/A	No	30 time points pre and post intervention each 2 consecutive days collected at ~7 a.m., at 1 p.m., and at 10 p.m. once per week during intervention collected at ~7 a.m., at 1 p.m., and at 10 p.m.	Average daily values.	None	Baseline salivary cortisol was higher in the control compared to MBSR-ld group. There were no changes in the average daily levels of salivary cortisol over time in both groups and there were no differences from pre to post intervention.	AS (?) AC (?) PK (?) IO (−)	Man (+) Trai (?) Inte (?)
Oken et al., 2010	31 caregivers. Mean age: 62.50 ± 11.60 years (Mindfulness), 67.09 ± 8.36 years (Education), 63.80 ± 7.92 years (Respite). Gender: male 19%, female 81% (total).	Mindfulness (based MBCT) (*N* = 10). 6 weeks + common first-week session (total 7 weeks) programme training: 10.5 h. Homework: not declared contents and lengths of time written material and recorded audio instructions.	Education (AC) (*N* = 11). 6 weeks + common first-week session (total 7 weeks) programme training: 10.5 h caregiver help book. Respite (PC) (*N* = 10) 7 weeks respite care: 3 h	N/A	No	6 time points pre and post intervention single day collected at within 5′ after awakening, 30′ later before eating, and at bedtime (~10–11 p.m.).	Each measured value (including morning levels).	IL-6, TNF-α, HS-CRP	There were no significant changes among the three groups in the levels of salivary cortisol.	AS (−) AC (−) PK (−) IO (?)	Man (+) Trai (+) Inte (?)
Jensen et al., 2012	47 healthy (mainly university students). Mean age: 20–36 years (total). Gender: male 34%, female 66% (total).	MBSR (*N* = 16) 8 weeks + intensive retreat (7 h) programme training: 27 h. Homework: formal assignments (45 min) following CDs with guided meditation practices and informal assignments (15 min).	NMSR (AC) (*N* = 15) 8 weeks (not declared intensive retreat) structurally similar to MBSR but did not include meditation practices or training in a non-judgemental attitude. Inactive controls (PC) (*N* = 16).	N/A	No	10 time points baseline and post intervention single day collected upon awakening, at 15, 30, 45, 60 min after awakening.	CAR: AUCG and AUCI.	None	The groups did not initially differ on any cortisol measures. At post intervention, MBSR group showed a tendency toward a lower AUCGthan did the inactive controls. MBSR group decreased near-significantly on AUCG, NMSR decreased, and the inactive controls increased within each group. Only MBSR decreased significantly on AUCI, NMSR decreased, and the inactive controls showed no changes.	AS (?) AC (?)PK (−)IO (−)	Man (+) Trai (+) Inte (?)
Flook et al., 2013	18 teachers. Mean age: 46.70 ± 6.95 years (MBSR-m), 38.50 ± 11.49 years (Control). Gender: male 11%, female 89% (total).	MBSR modified (*N* = 10) 8 weeks + one day-long immersion (6 h) programme training: 26 h. Homework: guided and unguided meditation practices that ranged in duration from 12 to 45 min guided practices following audio CDs.	Wait-list control (PC) (*N* = 8).	N/A	No	18 time points pre and post intervention each 3 consecutive working days collected at 30′ after awakening, before lunch, and before bed.	Morning levels and average daily values.	None	Both groups showed a marginally significant flattening of diurnal cortisol profiles over time. Although MBSR-m group didn't change the levels of morning salivary cortisol, the control group showed a significant decrease in the levels of that cortisol.	AS (?)AC (?) PK (?) IO (?)	Man (+) Trai (+) Inte (?)
Rosenkranz et al., 2013	49 community volunteers. Mean age: 44.4 ± 12.37 years (MBSR), 48.9 ± 7.66 years (HEP), Gender: male 20%, female 80% (total).	MBSR (*N* = 28) 8 weeks + one full-day session programme training: 20 h + α. Homework: daily at-home practice that ranged in duration from 45 to 60 min (not declared contents).	Health Enhancement Programme: HEP (AC) (*N* = 21) 8 weeks + one full-day session structurally similar to MBSR consisted of four components: (1) physical activity (2) balance, agility, and core strength (3) nutritional education and (4) music therapy.	4 months	Yes	18 time points: TSST pre and post intervention, and 4 months follow-up, single day collected after 20′ rest period, immediately before TSST, immediately after TSST, and subsequent 10′ intervals for 30′ during TSST. 45 time points: at home pre and post intervention, and 4 months follow-up each 3 days collected upon awakening, at 30′ post-awakening, before lunch, at 3 p.m., and before bed.	Diurnal slope and daily output (AUCGacross the whole day).	blister fluid TNF-α, IL-8	There was neither a significant effect of group, nor a group × time interaction for stress-evoked cortisol response. The slope of the decline in cortisol produced across the day did not differ between the two groups at pre intervention, whereas there was a non-significant trend for the slope to be steeper for MBSR group and less steep for HEP group at post intervention, that became significant at 4 months follow-up. Cortisol AUC showed a main effect of group, where MBSR had lower daily cortisol output across assessments, but no main effect of time or group × time.	AS (?) AC (?) PK (?) IO (?)	Man (+) Trai (+) Inte (?)

AC, active controls; AUC, area under the curve; AUC_G_, area under the curve with respect to ground; AUC_I_, area under the curve with respect to increase from awakening; BMI, body mass index; CAR, cortisol awakening response; CD, compact disc; HS-CRP, high-sensitivity C-reactive protein; IL, interleukin; MBCT, mindfulness-based cognitive therapy; MBSR, mindfulness-based stress reduction; mMBSR, modified mindfulness-based stress reduction; N/A, not available; NMSR, non-mindfulness stress reduction; Non-RCTs, non-randomized controlled trials; PC, passive controls; RCTs, randomized controlled trials; TNF-α, tumor necrosis factor-alpha; TSST, the Trier Social Stress Test. ST-Q: study quality. AS, adequate generation of allocation sequence; AC, concealment of allocation to conditions; PK, prevention of knowledge of the allocated intervention; IO, dealing with incomplete outcome data; St Q, Risk of bias (Higgins et al., 2011); Tx Q, Intervention quality; Man, the study referred to the use of a treatment manual; Trai, the therapists who conducted the therapy were trained; Inte, treatment integrity was checked during the study; +, high; −, low; ?, unclear.

In relation to interventions, two studies (Jensen et al., 2012; Rosenkranz et al., 2013) were performed with the standard MBSR programme (Kabat-Zinn, 1990), two were carried out with modified MBSR (Klatt et al., 2009; Flook et al., 2013), while the remaining one involved MBCT (Oken et al., 2010). The length of each intervention was 8 weeks in three studies (Jensen et al., 2012; Flook et al., 2013; Rosenkranz et al., 2013), 7 weeks in one study (Oken et al., 2010), and 6 weeks in another (Klatt et al., 2009). The total duration in hours of the training programmes ranged from 6 to 27 (mean: 17.90; *SD*: 9.34). One study did not declare the hours of one-full day session (Rosenkranz et al., 2013). The length of daily homework ranged from 12 to 60 min. One study did not state the length and contents of homework (Oken et al., 2010), while another study did not state the contents (Rosenkranz et al., 2013).

With respect to the assessments of salivary cortisol, the total number of measurements in each study ranged from 6 to 45 time points. One study (Klatt et al., 2009) measured the levels of salivary cortisol not only at pre and post-intervention, but also each week during the 6-week intervention. Another study (Rosenkranz et al., 2013) collected saliva samples in a test related to the Trier Social Stress Test (TSST), but also reported measurements in normal conditions (without a stress test). The total days of measurements of salivary cortisol ranged from 1 to 3 days: two trials (Flook et al., 2013; Rosenkranz et al., 2013) employed triple measures (i.e., 3 days); two trials (Oken et al., 2010; Jensen et al., 2012), a single measure (i.e., 1 day); and one (Klatt et al., 2009) double measures (i.e., 2 days). The total number of time point measurements per day were 3 in three trials (Klatt et al., 2009; Oken et al., 2010; Flook et al., 2013), and 5 in two trials (Jensen et al., 2012; Rosenkranz et al., 2013).

Of the included studies, only two used standard indicators: Rosenkranz et al. (2013) measured the levels of salivary cortisol using daily output and diurnal slope, and Jensen et al. (2012) using CAR. On the other hand, the other three studies (Klatt et al., 2009; Oken et al., 2010; Flook et al., 2013) assessed the levels of salivary cortisol using only raw data (average daily values or each measured value including morning levels). In terms of using a robust strategy for sample collection, only two trials (Jensen et al., 2012; Flook et al., 2013) measured salivary cortisol levels in accordance with established procedures, standardizing the time for sample collection, but also controlling for certain drinks and foods, and providing instructions on how to collect samples.

### Quality of included studies

According to the Cochrane Collaboration's tool for assessing risk of bias (Higgins et al., 2011), only one study (Oken et al., 2010) was considered as “high quality” (Table 3). With regard to the quality of the interventions, the use of a treatment manual was reported in all studies; therapist training was reported to be specific for the delivered intervention in four studies; and treatment integrity was verified in none. Therefore, none of the studies met all three criteria; four studies met two of the criteria; and one study did not meet any of the criteria for the quality of psychotherapy interventions (Chambless and Hollon, 1998; Table 3). Finally, only one study (Rosenkranz et al., 2013) used intention-to-treat data analysis.

### Outcomes of salivary cortisol

**Figure 2:**
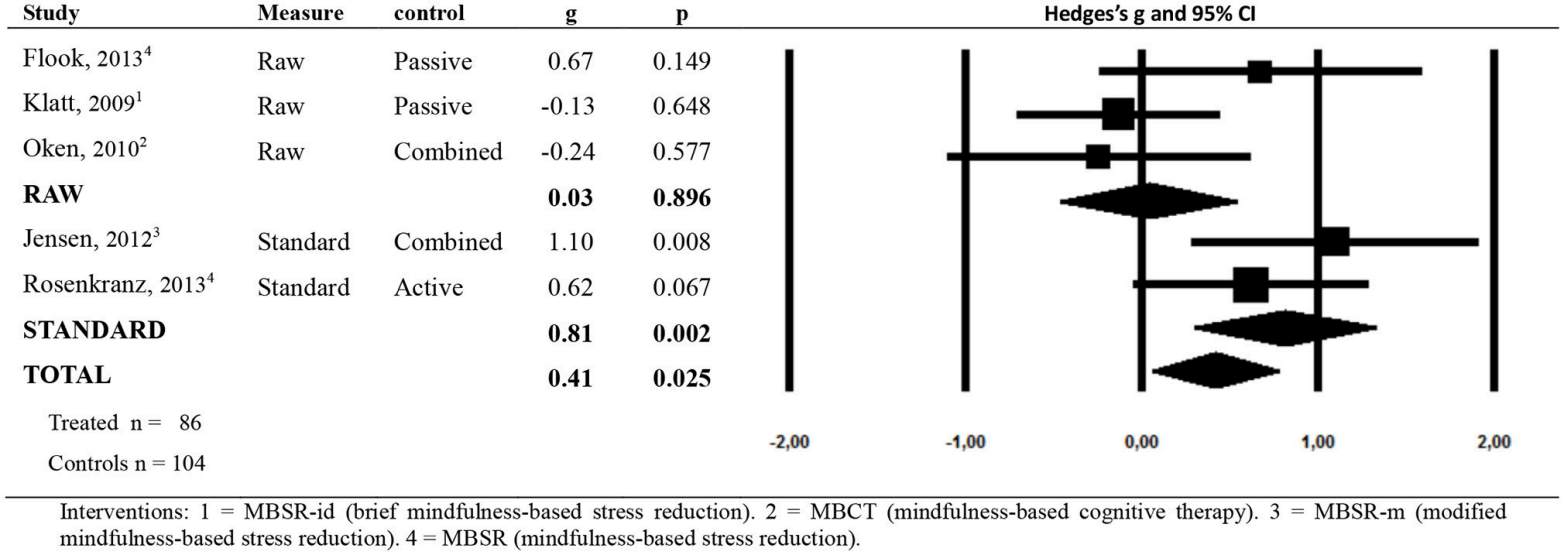
**Forest Plot for the overall effect size**.

As observed in Figure 2 and Table 4, and taking into account the criteria referred above, in general terms, MBIs showed moderately low effects in improving the state of health related to cortisol levels (*g* = 0.41; *p* = 0.025; NNT = 4.27), with moderate heterogeneity (*I*^2^ = 55; 95% CI = 0–83; *p* = 0.063). No indication of publication bias was found in the overall estimate (Begg *p* = 0.403; Egger *p* = 0.245). Therefore, it was not necessary to apply Duval and Tweedie's trim and fill procedure for imputing values. As shown in Table 4, the type of comparison group (AC vs. PC) did not explain heterogeneity, with moderate or moderately low ES values. On the contrary, the comparison according to the type of measure (raw vs. standard) showed significant differences in ES values. MBIs showed higher ES values using standard indices (*g* = 0.81; *p* = 0.002; NNT = 2.25), with low heterogeneity (*I*^2^ = 0; *p* = 0.374), than using raw data (*g* = 0.03; *p* = 0.896; NNT = 59.09), with moderately low heterogeneity (*I*^2^ = 23; *p* = 0.273). The percentage of women included in the study was not related to ES, and its explanatory power was null. However, the age of participants (beta = −0.03; *p* = 0.039; *R*^2^ = 0.80), number of sessions (beta = 0.33; *p* = 0.007; *R*^2^ = 1.00) and hours of programme (beta = 0.06; *p* = 0.005; *R*^2^ = 1.00) were significantly related to ES, explaining heterogeneity.

**Table 4:** **Effect sizes, heterogeneity and meta-regression**.

**Effects/heterogeneity**	***n***	***g***	**95% CI**	***p*^a^**	**NNT**	***I*^2^**
TOTAL	5	0.41	0.05–0.77	0.025	4.27	55
**COMPARISON**						
Active control	3	0.33	−0.18–0.84	0.202	5.26	27
Passive control	4	0.48	−0.39–1.35	0.279	3.6	79^*^
**MEASURE**^†^						
Raw	3	0.03	−0.46–0.53	0.896	59.09	23
Standard	2	0.81	0.30–1.33	0.002	2.25	0
**Meta-regression**	***n***	**Beta**	**95% CI**	***p*^b^**	***R*^2^**	***p*^c^**
% female	5	−0.02	−0.10–0.06	0.594	0.00	0.039
Age	5	−0.03	−0.06–0.01	0.039	0.80	0.284
Number of sessions	5	0.33	0.09–0.56	0.007	1.00	0.663
Hours of programme	5	0.06	0.02–0.09	0.005	1.00	0.834

n, number of included studies; g, Hedge's g effect size; 95% CI, confidence interval; p^a^, p-value associated with g; NNT, number needed to treat; I^2^, proportion of real observed dispersion.

* p-value associated to heterogeneity test < 0.01. Beta, coefficient of meta-regression; p^b^, p-value associated with the Beta coefficient; R^2^, proportion of total between-study variance explained by model; p^c^, p-value associated with the goodness of fit of the model assuming that the unexplained variance is null.

† Significant subgroup contrast at p < 0.05.

### Power calculation

Because of the limited number of studies found, a statistical power calculation was conducted to examine if we had taken into account a sufficient number of studies and sample sizes in order to identify relevant effects. This sensitivity calculation was conducted according to the procedures described by Borenstein et al. (2009). These calculations indicated that the inclusion of 5 studies, with a mean sample size of 38 (19 participants per condition), would allow a moderate effect size of 0.52 to be detected, assuming a conventional, moderate degree of heterogeneity (Hedges and Pigott, 2001), with a significance level alpha of 0.05, and with a fair statistical power of 0.78.

## Discussion

### Summary of findings

To our knowledge, this is the first meta-analytical review to explore the effects of MBIs on the levels of salivary cortisol in healthy adult subjects. Few studies examining the changes in salivary cortisol levels after MBIs have focused on this population. After a comprehensive literature search using four databases, we found five RCTs that fulfilled our inclusion criteria.

Our meta-analysis showed a significant moderately low effect for improving the state of health, based on cortisol levels, resulting from MBIs in healthy populations, with moderate heterogeneity and a low risk of publication bias. On the other hand, the sub-group and meta-regression analysis suggested possible differences in ES according to the type of measure used, and the age of participants, as well as a dose-response relationship between the hours and number of sessions of the programme, and the effect obtained. Taking the included studies independently, one trial (Jensen et al., 2012) showed obvious effects of MBIs on the levels of salivary cortisol, and another trial (Rosenkranz et al., 2013) showed marginal effects. These two trials used standardized measures of cortisol. In contrast, the other trials, which used raw cortisol data (Klatt et al., 2009; Oken et al., 2010; Flook et al., 2013), showed no significant efficacy of the MBIs.

### Interpretations of findings

There were three main possible factors that may have affected the findings across studies. The primary factor was the assessments of salivary cortisol, including the strategy for sample collection, total days of measurements and assessment indicators. There was a tendency to show more pronounced effects under conditions of using a structured strategy for sample collection, more days of measurements, CAR, daily output and diurnal slope as assessment indicators, not using only raw data (i.e., average daily values or each measured value including morning levels). In general, cortisol measurement, particularly salivary cortisol, requires that attention should be paid to the following points: (1) cortisol has a strong circadian rhythm, with levels peaking during the first hour after awakening, and decreasing for the rest of the day, with its nadir reached at around midnight; (2) saliva samples can be affected by numerous factors, such as food intake, smoking, caffeine consumption, rigorous exercise, and timing of collection (Matousek et al., 2010). Thus, Hanrahan et al. (2006) proposed: (1) standardizing the time for sample collection, including baseline samples; (2) using consistent collection materials and methods; (3) controlling for certain drinks, foods, medications, and diagnoses; and (4) establishing procedures and protocols. In this sense, Hellhammer et al. (2007) assessed the CAR in participants under real life conditions on six consecutive days and suggested that between 2 and 6 days were necessary to achieve reliable trait measures. On the other hand, with respect to outcome assessment, three indicators are generally used in assessing salivary cortisol, i.e., CAR, daily output, and diurnal slope. Our subgroup analysis showed that MBIs were more effective using standard indices than using raw data, with low heterogeneity (although the number of studies in this analysis was low). Future research should be implemented to assess salivary cortisol under the conditions of rigorous sample collection strategy, multiple days of assessments and adequate indicators.

Another factor was the contents of MBIs, specifically the total hours and sessions of training programmes. Our meta-regression analysis showed that the number of sessions and the total hours of programme duration were obviously associated with ES. In other words, there was a tendency to show more pronounced effects under conditions where interventions were conducted with a higher number of sessions and hours of training. Three trials (Jensen et al., 2012; Flook et al., 2013; Rosenkranz et al., 2013) were carried out with more than 20 h over 8 weeks. By contrast, the other two trials (Klatt et al., 2009; Oken et al., 2010) were performed with 6 and 10.5 h over 6 and 7 weeks, respectively. It was elucidated from the results that the former three studies (Jensen et al., 2012; Flook et al., 2013; Rosenkranz et al., 2013) seemed to have more effective impacts on salivary cortisol levels than the latter two studies (Klatt et al., 2009; Oken et al., 2010). Future research should be conducted on MBIs under conditions with more than 20 h of programmed training. Another important point is the type of MBI programme used (MBSR vs. MBCT). Only Oken et al. (2010) based their programme on MBCT, unlike the other studies which essentially used MBSR.

An additional factor was the characteristics of participants. The findings of our meta-regression analysis demonstrated that MBIs were more effective in younger subjects than in older participants, although there were no significant effects of MBIs on the proportion of women. A recent study (Harden et al., 2016) reported that the average salivary cortisol in older subjects (age 70–88), was significantly higher compared with younger adults (age 20–30). However, a previous review (Fries et al., 2009) noted that results in the relationships between salivary cortisol levels and age and gender are inconsistent. Although a recent meta-analysis has shown that mindfulness interventions are promising for youth (Zoogman et al., 2015), it is not clear how the age of participants might be related to the effects of MBIs on salivary cortisol levels. On the other hand, in some studies, participant profiles could have influenced baseline stress levels, and therefore baseline salivary cortisol, even though we only included healthy adult subjects to decrease possible variations and inconsistences. In this sense, one trial in particular (Oken et al., 2010) is noteworthy. Oken et al. (2010) carried out a pilot RCT with community-dwelling caregivers for close relatives with dementia. The subjects were required to be providing at least 12 h per week of assistance for the person with dementia, and they also had important stress levels, >9 on the Perceived Stress Scale (Cohen et al., 1983). O'Leary et al. (2016) pointed out that the latter criterion presupposed intervention utility in healthy subjects with high-stress only, and might result in greater decreases than usual. It was proposed that the high-stress baseline condition of each participant in this trial (Oken et al., 2010) could affect the changes in salivary cortisol levels between pre and post-intervention. In this study, Oken et al. (2010) also reported significant relationships between salivary cortisol levels with depression at post-intervention. A meta-analysis (Knorr et al., 2010), demonstrated that there was a small but significant mean difference between morning and evening salivary cortisol in patients with depression compared to controls, and therefore that there may be some association between the levels of salivary cortisol and depression scores. In contrast, regarding the relation between salivary cortisol and stress, one study was discarded (Nyklíček et al., 2013), and was not included in the meta-analysis because it only used stress-test measures. The inclusion of this study might have artificially inflated the general ES value by introducing a measure that was not comparable with the rest. It would be worthy of future research to conduct RCTs that paid more attention to the association between effects of MBIs on salivary cortisol and other psychological measures, including stress, pain, anxiety, burnout, and depression.

With regard to study design, especially the type of control group, our subgroup analysis showed no significant differences between ACs and PCs, although the trend seemed to be that MBIs might be more effective when using PC. Of the included studies, one trial (Rosenkranz et al., 2013) was carried out with an AC, while two of them (Oken et al., 2010; Jensen et al., 2012) with a two-arm control design (i.e., AC and PC), and another two (Klatt et al., 2009; Flook et al., 2013) with a PC. It was elucidated from the results that one study (Jensen et al., 2012) could show more useful effects on the levels of salivary cortisol than the others. It remains unclear whether the effectiveness of MBIs on salivary cortisol levels relates to the study design with AC or PC. In addition, the above-mentioned review (O'Leary et al., 2016) concluded that there were significant changes in the levels of cortisol in studies with a within-subject design but not in RCT designs, although they included participants with substance abuse, breast cancer, depression, and overweight/obesity. It is reasonable to think that future research may be recommended by means of RCTs conducted under AC conditions.

### Limitations

This systematic review and meta-analysis has several limitations. Firstly, few studies measuring changes in levels of salivary cortisol after MBIs have focused on healthy adult subjects. In fact, only five RCTs were included in the present meta-analytical review. Therefore, our findings should be interpreted with caution. However, we have seen that our sensitivity analysis showed enough statistical power in terms of the studies and samples sizes found. Secondly, we found different strategies for sample collection, days of salivary cortisol measured, and indicators to assess possible changes in cortisol. We also found different protocols for MBIs, number of sessions, and hours of programmed training. In this sense, heterogeneity was moderate, showing that the results across studies could take different values according to their own characteristics. Lastly, another possible weakness is related to the unclear quality of most of the studies. Our results suggest no important publication bias. However, it might be reasonable to expect more attenuated effects, especially considering that the overall quality of studies, according to the reported data, did not seem very high.

## Conclusions

Results from our meta-analysis indicate that MBIs may have a beneficial effect on salivary cortisol secretion in healthy adult subjects, and that effect seems to be dependent of the total hours of training, number of sessions and characteristics of participants such as age. However, the observation of effects might be influenced by the type of cortisol measure used, although there is a paucity of robust evidence to enable a conclusion to be reached, given the scarce number of studies. Future research protocols concerning MBI implementation focused on salivary cortisol changes in healthy population should consider: (1) salivary cortisol should be measured adhering to a rigorous strategy or protocol for sample collection, using multiple days of measurements and appropriate indicators (i.e., CAR, daily output and diurnal slope); (2) MBIs should be performed in accordance with standard programmes such as MBSR or MBCT, particularly regarding the total sessions and hours of training included in the programme; (3) study designs should favor the use of RCTs with AC interventions, and reporting of all the aspects of the quality of studies and interventions seems mandatory.

## Author contributions

KS, JM, MD, MG, and JG designed the project. MP, HM, MS, and MA collected the data. JM performed the statistical analysis. All authors interpreted the results, drafted the manuscript and read and approved the final manuscript.

### Conflict of interest statement

Within the past three years, KS has received speaker's fees from Janssen Pharmaceutical. This sponsor had no influence on this study and the present work was not supported by any funding. The other authors declare that the research was conducted in the absence of any commercial or financial relationships that could be construed as a potential conflict of interest.
